# Chief Executive Officer Proactive Personality and Acquisitions: A Fuzzy Set Qualitative Comparative Analysis of China's Listed Firms

**DOI:** 10.3389/fpsyg.2021.703678

**Published:** 2021-08-09

**Authors:** Gang Yang, Xueyan Bai, Shiyu Yang

**Affiliations:** ^1^School of Management, Jilin University, Changchun, China; ^2^School of Economics and Management, Tongji University, Shanghai, China

**Keywords:** proactive personality, CEO characteristics, M&As, fsQCA, emerging industries, upper echelons theory

## Abstract

The role of the CEO in an enterprise's management decisions renders their individual characteristics influential in decisions about mergers and acquisitions (M&As). Personal characteristics are based on many aspects, therefore, we provide a multi-angle insight into the personal characteristics of managers. Drawing on the upper echelons theory, we examine whether CEOs' proactive personality affects merger and acquisition decisions. The fuzzy-set qualitative comparative analysis (fsQCA) is performed using a sample of 64 listed firms in China for the period 2010–2019. There are three solutions for cross-industry mergers, and five for intra-industry mergers. The results suggest that: (a) proactive and overconfident CEOs are inclined toward cross-industry mergers; (b) non-proactive and low-educated CEOs are inclined toward intra-industry mergers; (c) emerging industry enterprises tend to choose intra-industry mergers; (d) overconfident CEOs are more likely to undertake cross-industry mergers in traditional industries.

## Introduction

Mergers and acquisitions (M&As) are typically strategic decisions in business management; they have become an important way for enterprises to realize rapid scale expansion and development (Lee and Lieberman, 2010). In this vein, the Upper Echelons Theory (UET) advances that the decisions and behavior of its executives are largely dependent on their socio-demographic features and psychological variables.

Previous empirical studies have broadly examined the relationship between CEO characteristics and M&A decisions. For example, Ferris et al. (2013) focused on the overconfidence of CEOs. They found that overconfidence helped to explain the number of offers made by a CEO, the frequencies of non-diversifying and diversifying acquisitions, and the use of cash to finance a merger deal. In terms of managerial background, research suggested that CEOs with financial experience would choose aggressive business strategies (Custódio and Metzger, 2014). Thanks to the cross-fertilization between psychology and upper echelons theory, scholars have increasingly been considering the influence of personality traits of executives on decision-making, such as narcissism, hubris, or overconfidence (Ham et al., 2018; Malhotra et al., 2018; Abatecola and Cristofaro, 2020). In fact, the proactive personality of executives has also a significant influence on strategic decisions, especially on M&A decisions. Proactive personality is the tendency of individuals to take active actions to change their external environment (Bateman and Crant, 1993). Contrasting with intra-industry mergers, cross-industry mergers will bring more changes and challenges from the external environment for executives. On this basis, it is logical to anticipate: the CEOs of enterprises that choose cross-industry mergers are more likely to have proactive personality. In doing that, our study analyzed the comprehensive influence of different characteristics of CEOs on M&A decisions and incorporated socio-demographic and psychological features of CEOs into a unified research framework.

Yet, over time the evolving context of upper echelons analyses has obtained widespread attention, especially for a number of research implications in terms of firm-environment relationships (Abatecola and Cristofaro, 2020). Looking at the industry level of the environment, the most investigated industries have been those of semiconductors, furniture, food, aerospace, and cement (Abatecola and Cristofaro, 2020). Hambrick and Quigley (2014) examined the industry's discretion and analyzed the industry's condition through the size-weighted mean return on assets. However, in a multitude of studies on mergers and acquisitions, the industry is usually used as a control variable (Lin et al., 2018). In fact, the impact of industry characteristics on M&As cannot be ignored. Hence, our study analyses the influence of industry on M&A decisions. Based on the industry division in China's 13th Five-Year Plan for the Development of National Strategic Emerging Industries, our study introduces emerging industries and traditional industries as conditions and explores the difference in the choice of M&As between them.

Based on the above, we explore the influence of different factors on M&A decisions, including socio-demographic features of CEOs, psychological variables of CEOs, and industry level of the environment. In our study, CEO proactive personality, overconfidence, educational background, financial experience, and industry are taken as conditions, and the fuzzy set qualitative comparative analysis (fsQCA) is used for the sufficiency and necessity of the conditions. The influences of multiple conditions on the results are comprehensively explained from the perspective of configuration (Fiss, 2011). Through the fuzzy-set qualitative comparative analysis (fsQCA) method, our study explores not only the core conditions that affect M&A decisions but also the marginal factors when many characteristics appear in the same manager. In addition, the rapid increase of M&As in China provides the context within this study. Different from global M&A waves, which started at the end of the 19th century and experienced more than a century, Chinese M&As started late but developed rapidly in the last 20 years. Currently, China's economy is in a transition stage, with the profitability of traditional industries in decline. Many enterprises seek new profit growth points through M&As to transform or expand traditional businesses. Therefore, our study selected China as a context and used 64 M&As of Chinese listed companies from 2010 to 2019 as examples for empirical analysis.

Our study contributes significantly to the existing literature. Firstly, it adds to the literature on proactive personality and presents novel evidence on how CEO proactive personality affects M&A decisions. The results show that proactive CEOs are more inclined toward cross-industry mergers. Secondly, through the fsQCA, M&A decisions are shown to be influenced by several conditions simultaneously. The results of the QCA of the fuzzy sets show that there are three configurations for cross-industry mergers and five paths for intra-industry mergers. Finally, this study introduces traditional and emerging industry conditions and finds that the type of the acquirer's industry has a significant influence on the choice of M&A target. The results show that intra-industry mergers occur more in emerging industries. Furthermore, in intra-industry mergers, CEOs are usually characterized by a focus on the industry, low innovation, and low education. However, in cross-industry mergers, CEOs are usually characterized by proactive personality and overconfidence.

The remainder of this study is organized as follows. Section Theoretical Framework and Literature Review reviews the related literature and theories. Section Context of the Research introduces the context of the research. Section Research Design describes our data and the fsQCA method. Section Research Results shows empirical analysis. Finally, we discuss the results and summarize the main conclusions of the paper.

## Theoretical Framework and Literature Review

### Upper Echelons Theory

The upper echelons theory holds that the decisions and behavior of the enterprise are realized by the decisions and behavior of its senior executives, which in turn are largely dependent on their psychological and demographic characteristics (Hambrick and Mason, 1984). The characteristics, corporate governance, or investment decision-making research in the literature mainly focuses on three aspects: The first is the relationship between managers' experience and corporate governance performance (Wang and Yin, 2018; Burns et al., 2021). The second is the relationship between managers' personality and corporate governance or operating performance (Billett and Qian, 2008; Malmendier and Tate, 2008). The third is the relationship between executives and corporate governance or operating performance (Shi et al., 2019; Wang et al., 2020).

After that, many studies have continuously improved the theory, including proposing the job requirements for senior executives as a moderator variable (Hambrick, 2007). The personal ambition of a manager in the job requirements is presented as a desire for success and self-actualization. High job requirements drive managers to take shortcuts and to rely on their previous successful experiences; in turn, their previous experience will have a stronger influence on their decisions (Jeganathan et al., 2021).

Existing literature on the influence of managers' personality traits on M&As focuses on overconfidence, extraversion (Malhotra et al., 2018), and pre-existing narcissism (Ham et al., 2018). Recently, researchers investigated the impact CEOs' dispositional preventative focus had on firms' deal structuring choices in M&As (Gada et al., 2021). Additionally, Shi et al. (2019) verified that in the presence of high CEO-CFO language style matching, firms tended to undertake more mergers and acquisitions. Chen et al. (2021) found that the fit between CEO human capital makeup and their type of acquisitions relates to stronger performance. Moreover, studies exist on the educational background (Wang and Yin, 2018), social status (Plaksina et al., 2019), executive migration (Wang, 2019), supply chain industry experience (Burns et al., 2021), and multiple merger experience (Jeganathan et al., 2021). Acquisitions by firms with high managerial ability generated better abnormal returns at the announcement as well as better post-announcement abnormal returns than firms with low managerial ability did (Chen and Lin, 2018). Bachmann and Spiropoulos (2021) proposed that bidders with female board members preferred to target firms that also have female board representation.

### CEO Proactive Personality

Based on interactionism, proactive personality is defined as the tendency of individuals to take initiative to change their external environment (Bateman and Crant, 1993). Studies show that proactive personality has a positive effect on job performance (Wei et al., 2021) and work attitude (Harvey et al., 2006), leadership transitions (Lam et al., 2018), entrepreneurial intention (Baluku et al., 2020), sustainable investment (Vanwalleghem and Mirowska, 2020), employee creativity (Li et al., 2021), and employee resilience (Zhu and Li, 2021).

Through the influence of proactive personality, executives tend to change the existing environment when making decisions. M&As obviously pose new challenges to the existing business environment. However, proactive personality has not been introduced into M&A research.

Regarding the measurement of proactive personality, Bateman and Crant (1993) first developed a 47-item questionnaire. Through exploratory factor analysis, a 17-item proactive personality scale was obtained; subsequent research reduced the scale to 10 items (Seibert et al., 1999). In addition, some studies further reduced the proactive personality scale to 6 items (Parker, 1998), 5 items (Kickul and Gundry, 2002), and 4 items (Parker and Sprigg, 1999). Table 1 summarizes the core information for proactive personality. To demonstrate the cross-cultural universality of the proactive personality scale, Claes et al. (2005) used samples from Belgium, Finland, and Spain. Based on the characteristics of China's economic system, corporate governance, and cultural environment, the scale used to measure a proactive personality may not be suitable for cross-cultural Chinese samples. Therefore, the use of the proactive personality scale measurement that has been carried out by previous researchers is doubtful in this study and cross-cultural consistency of the proactive personality scale remains to be tested.

**Table 1:** Illustration of proactive personality and overconfidence.

**Type**	**Proactive personality**	**Overconfidence**
Theory	Interactionism	Behavior finance theory
Concept	Tendency to change the external environment	Cognitive bias
Measurement	17-item proactive personality scale; 10-item scale; 6-item scale; 5-item scale; 4-item scale	CEO shareholding; CEO relative compensation; Historical business performance; Frequency of CEO M&As; Weight of manager personal characteristics; Business climate index; CEO evaluation by mainstream media; Earnings forecast bias; CEO being a founder or heir
Outcome	Job performance; Work attitude; Leadership transitions; Entrepreneurial intention; Sustainable investment; employee creativity; Employee resilience	Ambidextrous innovation Diversification Bigbaths Overinvestment Firm risk

The above literature focuses on the influence of individual proactive personality on individuals and organizations, rather than on decision-making; there has been no in-depth research on the relationship between proactive personality and M&As in the literature related to developments in the upper echelons theory. Our study complements the existing literature on proactive personality and corporate strategy.

### CEO Overconfidence and M&As

The overconfidence of managers is one of the factors that have been widely studied in M&A decision-making. Overconfident managers tend to have psychological and cognitive biases. They will ignore risks, overestimate their own abilities, and make irrational decisions (Brown and Sarma, 2007). It is precise because managers have more rights to make important decisions and choices than ordinary employees that they tend to show overconfidence (Hayward and Hambrick, 1997). Overconfidence in M&A decisions was first studied by Roll (1986). This was followed by the widely recognized study on M&As by Malmendier and Tate (2008). Their study found that overconfident managers are 65% more likely to carry out M&A activities than rational managers. When the internal capital of an enterprise is abundant and the executive has the mandate to undertake diversifying mergers, it is obvious that overconfident managers will undertake more M&As than rational managers. However, other studies suggest that, although overconfidence can promote M&As, it does not affect whether they are diversified (Ferris et al., 2013). CEO overconfidence is relatively mature in the study of M&As, although previous works have only done univariate studies.

CEO overconfidence is also widely used in other management research, including ambidextrous innovation (Wong et al., 2017), diversification (Andreou et al., 2019), big baths (Pierk, 2021), overinvestment (Kwon et al., 2021), and firm risk (Ali and Tauni, 2021). Many quantitative indicators have been used in the measurement of CEO overconfidence in the literature; for example, CEO shareholding (Malmendier and Tate, 2008), the relative compensation of CEOs (Huang et al., 2011), historical business performance, frequency of CEO M&As (Doukas and Petmezas, 2007), weight of manager personal characteristics (Barber and Odean, 2001), business climate index (Yu et al., 2006), CEO evaluation by mainstream media (Malmendier and Tate, 2008), and earnings forecast bias (Lin et al., 2008). However, in many of the above examples, it is not obvious when specific indicators are appropriate for the measurement. According to the control illusion theory (Langer, 1975), psychological research has found that people tend to believe that they can influence some random events. When people expect certain outcomes, and those outcomes do occur, they tend to attribute them to their actions rather than luck, and further confirm their control over the situation. Hayward and Hambrick (1997) found that the higher the relative salary of a CEO, the more likely it was to cause the illusion of personal control, resulting in managerial overconfidence. Huang et al. (2011) used this method to test Chinese companies and found the moderating effect of managerial overconfidence on the sensitivity of cash flows. Table 1 summarizes the core information for overconfidence.

### Managerial Background and M&As

Executives' background is also an important factor in M&A decision-making, according to the upper echelons theory. The background of an executive determines the decision-making horizon. A person's career is influenced by their past life experience; their education and past working environment will affect their future career behavior (Xie, 2003). There is a close relationship between the choice of intra-industry mergers or cross-industry mergers in the M&A decision and educational background and working experience. Some studies suggest that highly educated managers have a greater tolerance for ambiguity and uncertainty. They are more likely to accept the impact of environmental changes and formulate strategies conducive to enterprise reform and development (Bantel, 1993). Compared with CEOs with a low education level, CEOs with a high education level have more advantages in information processing. Many innovative and developmental enterprises are mostly led by managers with high education levels (Wally and Baum, 1994). CEOs with financial business experience have both a financial knowledge foundation and rich practical experience. They know how to use financial leverage to formulate business decision strategies rationally and to improve the profitability of enterprises (Jiang et al., 2012). In addition, managers with financial experience are better able to use professional financial knowledge to conduct capital operations and deal with crises, which affords them more confidence and tolerance for risks (Graham et al., 2013). CEOs with financial experience will choose aggressive business strategies (Custódio and Metzger, 2014). In terms of M&As, it has been shown that CEOs who obtained an MBA degree after the 1970s focus more on non-diversified acquisitions (Jung and Shin, 2019). Other studies believe that relevant experience of the target industry will promote the evaluation of the target company in the diversified M&As (Wang et al., 2015). CEOs with a broader set of knowledge and skills are more likely to engage in unrelated acquisitions (Chen et al., 2021).

Briefly, the choice of M&A target is the result of many factors. However, it is unknown how factors such as CEO proactive personality, overconfidence, and background work together to influence that choice. Traditional regression analysis methods have been used to explore the influence of a single key variable on the selection of merger type; however, the methods cannot effectively reveal the interaction between different variables (Fiss, 2011). Therefore, this study introduces the QCA method to explore the joint effect of the above three characteristics of a CEO on the choice of merger type. There exists abundant research on CEO overconfidence and demographic characteristics; combined with the improvements in the upper echelons theory, this study adds the characteristics of CEO proactive personality to reflect the influence of CEO ambition on the choice of merger types. Based on the characteristics of China's economic system, corporate governance, and cultural environment, it is meaningful to study Chinese M&A cases.

## Context of the Research

In the early stage of China's economic development since 2002, Chinese enterprises increased industrial concentration through mergers and acquisitions (M&As), to improve corporate competitiveness. At the same time, the Chinese government issued a series of laws and regulations on enterprise M&As and improved related market mechanisms. In 2005, China began non-tradable shares reform. The capital market continues to mature; the government has increased the structural adjustment of strategic corporations by proposing relevant financing policies. These actions promoted mergers and acquisitions and in the 2010s, they increased rapidly. M&As of Chinese enterprises increased from 2,947 in 2010 to 4,498 in 2019; in monetary terms, M&A transactions increased from USD143.9 billion to USD272.4 billion (PWC, 2014, 2019). M&A transactions in China's economic transformation usually have the following characteristics: (1) cross-industry M&As, wherein a layman manages an expert, (2) a traditional industry enterprise merging with an emerging enterprise, (3) a high P/E ratio enterprise merging with a low P/E ratio enterprise, (4) more attention on the M&A than on integration, and (5) the integration of only economies of scale. Shenwan Hongyuan's group chief of operations (CO) calls such M&As “Chinese-style merger and acquisitions.” With the rapid development of digitalization in China, acquisitions target the information technology, biomedicine, and chemical industries. The acquirers are not only digital companies, but also traditional corporations undergoing digital transformation, including those in the automobile, consumer goods, medical, retail, media, telecommunications industries as well as public utilities.

## Research Design

### Samples and Data

The M&A transactions of Chinese listed companies come from the RESSET database, which is the main event database for companies in the RESSET stock database. The data on innovation and social capital network of CEO proactive personality and CEO overconfidence is also from the RESSET database, whereas the background information on CEOs, which includes educational background and financial experience, comes from the CSMAR database. We require that (i) only the completed M&As are included, (ii) the merging firms are not in the financial industry, given differences in financial reporting systems, and related regulations, (iii) according to the purpose of M&As, leveraged buyouts, spin-offs, capital structure changes, privatization, and similar types of transactions be excluded, and (iv) the equity proportion in M&A transactions should not be <50%, to avoid portfolio investment situations. Following these criteria and eliminating observations with missing data, we collected 64 major Chinese domestic M&As during 2010–2019. Table 2 Panel A provides the sample selection process.

**Table 2:** Sample selection and distribution.

			**Total**
**Panel A: Sample selection**			
Mergers and acquisitions from Chinese mainland during			17,126
2010–2019			
*Less*			
Observations not complete M&As			1,908
Observations in the financial industry			2,198
Leveraged buyouts, spin-offs, capital structure changes,			8,513
privatization			
Observations equity proportion <50%			2,106
Observations with missing CEO data			2,337
Final sample			64
**Types**	**Industry**	***n***	**(%)**
**Panel B: Observations by merger type**			
Cross-industry merger	Emerging industry	20	31.25%
	Traditional industry	6	9.38%
Intra-industry merger	Emerging industry	30	46.88%
	Traditional industry	8	12.50%
Total		64	100.00%
**Types**	**Industry**	***n***	**(%)**
**Panel C: Observations by industry**			
Emerging industry	New-generation information technology	3	4.69%
	High-end equipment manufacturing	14	21.88%
	New materials	14	21.88%
	Biological	6	9.38%
	New energy automobile	2	3.13%
	New energy	1	1.56%
	Energy conservation and environmental protection	1	1.56%
	Digital creative	1	1.56%
	Related services	8	12.50%
Traditional industry		14	21.88%
Total		64	100.00%

Table 2, Panel B shows the breakdown of observations by M&A type. The results show that the most heavily represented M&A type is intra-industry mergers in emerging industries (46.88%), followed by cross-industry mergers in emerging industries (31.25%). The fewest observations are cross-industry mergers in traditional industries (9.38%).

Panel C presents the breakdown of observations by industry. The enterprises in the sample are divided according to the concept of strategic emerging industries proposed in China's 13th Five-Year Plan for the Development of National Strategic Emerging Industries. There are 50 enterprises in emerging industries, accounting for 78.12%. The remaining 14 are traditional industries. There are nine types of emerging industries: new-generation information technology industry, high-end equipment manufacturing industry, new materials industry, biological industry, new energy automobile industry, new energy industry, energy conservation and environmental protection industry, digital creative industry, and related services industry. The buyers of emerging industries in the sample are mainly in the high-end equipment manufacturing industry (14) and the new materials industry (14).

### Measurement

Table 3 shows the description, codification, and data source of the outcome and conditions. The choice of M&A target, *MEG*, is an outcome. Referring to the guidance on industry classification of listed companies issued by the China Securities Regulatory Commission (2013), different category codes are represented as A, B, C, etc. The large class code is represented by two numbers, coded sequentially, starting at 01. If all three codes of the acquiring enterprise and the target enterprise are identical, *MEG* equals 0. If they are not identical, *MEG* equals 1, which is regarded as a cross-industry merger.

**Table 3:** Outcome and conditions: description, codification, and data source.

	**Symbol**	**Description**	**Codification**	**Data source**
**Outcome**				
Merge type	*MEG*	A binary variable that assumes a value of 1 if the industry code of the buyer and the target enterprise are not the same, and zero otherwise	Crisp value	RESSET
**Condition**				
Proactive personality	*PRO_CP*	A binary variable that assumes a value of one if a CEO has a concurrent post in other companies, and zero otherwise	Crisp value	RESSET
	*PRO_R&D*	R&D investment divided by operating revenue	Fuzzy value	RESSET
Overconfidence	*OC*	Top three directors' salary divided by the total board of directors' salary	Fuzzy value	RESSET
Managerial background	*MB_EDU*	A binary variable that assumes a value of 1 if the highest degree of CEO is master's degree or above, and zero otherwise	Crisp value	CSMAR
	*MB_FIN*	A binary variable that assumes a value of 1 if CEO has ever worked in the financial department and financial analysis, and zero otherwise	Crisp value	CSMAR
Industry	*industry*	A binary variable that assumes a value of 1 if the industry of the enterprise is one of the nine emerging industries, and zero otherwise	Crisp value	RESSET

CEO proactive personality is an antecedent condition. The board of directors is the highest authority in listed companies in China. In the power structure and configuration of companies, the chairperson is the core of power and has the final say in decisions. Therefore, “CEO” in this study refers to the chairperson of the board. Some studies in psychology have found that proactive personality is significantly positively related to innovation (Kim, 2019) and social capital network in careers (Yang et al., 2011). Therefore, the chairperson's innovation and social capital network are used as proxy variables for proactive personality. The social capital network is measured by whether the CEO has a concurrent post in other companies (*PRO_CP*), which is regarded as a binary variable: Where they have a concurrent post, it equals 1, and 0 otherwise (Yang et al., 2011). The ratio of R&D investment divided by operating income measures innovation (Howella et al., 2020).

CEO overconfidence is an antecedent condition. As mentioned above, in the study of the illusion of control it is believed that the higher the compensation ratio of managers, the stronger their control. Therefore, the higher the managers' salary relative to that of the other managers in the company, the higher the managers' status, and the more likely they are to be overconfident. Considering the availability and feasibility of the data, the proportion of the top three directors' salary divided by the total board of directors' salary is used to measure CEO overconfidence (Jiang et al., 2011).

CEO background is an antecedent condition. Highly educated CEOs with MBA degrees are more likely to adopt aggressive and high-risk investment strategies (Bertrand and Schoar, 2003). Here, *MB_EDU* equals 1 if the CEO has a master's degree or above, and 0 otherwise. A CEO's financial experience is defined as having worked in a financial department or financial analysis; such a CEO has worked in an accounting or auditing position, has been in a finance or major finance position, has a license for a middle or senior accountant, or is a certified public accountant. CEO financial experience (*MB_FIN*) is regarded as a binary variable. If CEO has financial experience, it equals 1, otherwise, it is 0 (Jiang et al., 2012).

Emerging industries is also a binary variable, based, as mentioned above, on China's 13th Five-Year Plan for the Development of National Strategic Emerging Industries. There are nine types of emerging industries. The variable, *industry* equals 1 if a company is from an emerging industry, and 0 otherwise.

### Qualitative Comparative Analysis and Calibration

The fuzzy-set qualitative comparative analysis (fsQCA) is performed in our study. This method is moving beyond qualitative and quantitative strategies (Ragin, 1987). The primary function of the statistical method assumes that the relationship exhibits constancy, consistency, additivity, and symmetry. The collinearity between variables should also be strictly controlled. This assumption and the request in social science research are too idealistic. The QCA verifies the necessity and sufficiency of a single condition or conditional configuration by means of the relation between sets. In the relation of necessary conditions, the conditions constitute the superset of the result, without which the result cannot exist. In the relation of sufficient conditions, the conditions constitute a subset of the results, and the existence of the conditions can fully produce the results (Ragin and Fiss, 2008). The QCA method can be applied to cross-case comparisons of the large, medium, and small samples, especially in studies of small and medium samples (<100) (Fiss, 2011). In QCA, because of the logic of causal asymmetry, the conditional configuration (CC) affects the results from positive research; we can also further compare the configuration with the conditional one that leads to the disappearance of results, and the configuration obtained by the two kinds of analyses may be different.

The QCA includes three basic categories: clear set QCA (csQCA), A fuzzy set qualitative comparative analysis (fsQCA), and multi-value set QCA (mvQCA). Compared on the basis of characteristics, csQCA and mvQCA are only suitable for dealing with categorical problems, fsQCA can further deal with problems related to degree variation or partial membership. The fuzziness of granules, their attributes, and their values are characteristic of the ways in which humans granulate and manipulate information. Moreover, no methodology other than fuzzy logic provides machinery for fuzzy information granulation.

In fsQCA, each condition (i.e., the six factors in this study) and outcome (the choice of M&A target) are treated as a set. Each case has a membership score in these sets. The process of assigning membership grades is calibration (Schneider and Wagemann, 2012). Calibrated scores ranged from 0 to 1, representing cases without and with full membership, respectively. According to the data types of the various conditions and results, we use the direct calibration method (Ragin and Fiss, 2008) to convert the data into fuzzy set membership scores. The calibration process was based on the thresholds for full membership (≥0.75), no membership ( ≤ 0.25), and the crossover point (0.5). Table 4 summarizes the calibration information for each condition and outcome in this study.

**Table 4:** Calibration for outcome and conditions.

**Outcome and conditions**	**Calibration**		
	**Full membership**	**Cross-over point**	**Full non-membership**
*MEG*	1		0
*PRO_CP*	1		0
*PRO_R&D*	3.5175	2.595	1.11
*OC*	33.5725	28.225	25.2225
*MB_EDU*	1		0
*MB_FIN*	1		0
*industry*	1		0

## Research Results

### Necessary Condition Analysis

First, we examine whether a single condition (including its non-set) constitutes a necessary condition for a cross-industry merger or an intra-industry merger. Considering a set, the necessary analysis of a single condition is to check whether an outcome set is a subset of a set of conditions. In fsQCA, when an outcome occurs, a certain condition always exists; this condition is a necessary condition for the outcome (Ragin and Fiss, 2008). Consistency is an important criterion to measure the necessary condition. When the consistency level is higher than 0.9, the condition can be considered as a necessary condition for the outcome (Schneider and Wagemann, 2012).

Table 5 shows the test results for the necessary conditions for cross-industry mergers and intra-industry mergers, analyzed by fsQCA3.0 software. In Table 5, the outcome and all the conditions have been calibrated (suffix “fz” denotes a calibrated variable). The consistency level for all the conditions is not higher than 0.9; therefore, of the five conditions, none is a necessary condition for cross-industry mergers or intra-industry mergers.

**Table 5:** Analysis of necessary conditions.

**Condition**	**Cross-industry merger**		**Intra-industry merger**	
	**Consistency**	**Coverage**	**Consistency**	**Coverage**
*PRO_CP*	0.5769	0.4286	0.5263	0.5714
*~PRO_CP*	0.4231	0.379	0.4737	0.6207
*PRO_R&D_fz*	0.6954	0.5696	0.3595	0.4304
*~ PRO_R&D_fz*	0.3046	0.2455	0.6405	0.7545
*OC_fz*	0.7154	0.5730	0.3647	0.4270
*~ OC_fz*	0.2846	0.2346	0.6353	0.7654
*MB_EDU*	0.8462	0.4783	0.6316	0.5217
*~ MB_EDU*	0.1538	0.2222	0.3684	0.7778
*MB_FIN*	0.2308	0.3158	0.3421	0.6842
*~ MB_FIN*	0.7692	0.4444	0.6579	0.5556
*industry*	0.7692	0.4000	0.7895	0.6000
*~industry*	0.2308	0.4286	0.2105	0.5714

### Conditional Configuration Analysis of Cross-Industry Mergers

Conditional configuration analysis solves the problem of the sufficiency of an outcome caused by different configurations formed by multiple conditions. From the perspective of set theory, conditional configuration analysis checks whether a set constituted by multiple conditions is a subset of an outcome set. Consistency is still used in conditional configuration analysis to measure configuration sufficiency. The acceptable minimum criteria and calculation methods are different from those in conditional configuration analysis. Generally, the consistency of sufficiency is determined to be no lower than 0.75 (Schneider and Wagemann, 2012). In different research contexts, different consistency thresholds apply, such as 0.75 (Ragin and Fiss, 2008) and 0.8 (Fiss, 2011). The frequency threshold needs to be determined based on sample size (Schneider and Wagemann, 2012). For medium and small samples, the frequency threshold is usually 1; for large samples, the frequency threshold should be >1. Coverage is an important indicator that measures relevance in QCA and reflects the relevance or importance of a configuration. Coverage is similar to R^2^ in regression analysis (Fiss, 2011).

Having studied the truth table and the case, we set the consistency threshold to 0.75 and the frequency threshold to 1. Thus, the threshold setting includes at least 75% of the observations and reduces the potential conflict configuration, PRI (proportional reduction in inconsistency). However, there is no consensus or theoretical expectation on the relationship between the six conditions and cross-industry mergers or intra-industry mergers. Therefore, we choose “presence or absence” for the question of which state of the six conditions will lead to cross-industry mergers or intra-industry mergers (Schneider and Wagemann, 2012).

The software fsQCA3.0 outputs three solutions: a complex solution, a parsimonious solution, and an intermediate solution. We report intermediate solutions (Fiss, 2011), supplemented by parsimonious solutions (Fiss, 2011). Following Fiss (2011), solid circles (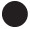) indicate the existence of a condition, crossed-out circles (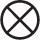) indicate the absence of a condition, and blank spaces indicate an ambiguous state. An ambiguous state means that a condition either exists or does not. A large circle signifies a core condition that exists in both the parsimonious solution and the intermediate solution. A small circle signifies an auxiliary condition (one that exists only in the intermediate solution). Core elements are those causal conditions for which the evidence indicates a strong causal relationship with the outcome of interest; and peripheral elements are those for which the evidence for a causal relationship with the outcome is weaker (Fiss, 2011).

There are five configurations with three solutions in Table 6. The consistency level for both the single configuration and the overall solution is higher than the acceptable minimum standard of 0.75. The consistency for the overall solution is 0.91, and the coverage is 0.40. These are consistent with the QCA research in the field of organization and management. The analysis shows that 91% of the cases satisfying these three solutions can lead to cross-industry mergers, while the three solutions can explain 40% of cross-industry merger transactions. The consistency level was adjusted from 0.75 to 0.8 for robustness tests, and the case frequency was changed from 1 to 2. The conclusion remained robust.

**Table 6:** Configurations leading to cross-industry mergers.

**Antecedent conditions**	**Cross-industry merger** (**presence of the outcome)**				
	**1a**	**1b**	**1c**	**2**	**3**
*PRO_CP*	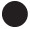	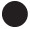	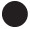	⊗	⊗
*PRO_R&D_fz*	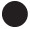	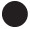	●	⊗	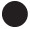
*OC_fz*	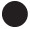	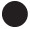	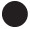	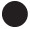	⊗
*MB_EDU*	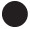	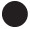		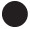	⊗
*MB_FIN*	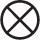		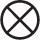	⊗	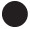
*industry*		●	●	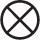	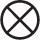
Consistency	0.8966	0.8552	0.8693	0.9135	1
Raw coverage	0.22	0.2135	0.1688	0.0365	0.0196
Unique coverage	0.0896	0.0831	0.0385	0.0365	0.0196
Solution consistency	0.9094				
Solution coverage	0.3977				
Cases coverage	7	7	6	1	1

*Solid circles (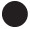) indicate the existence of a condition, crossed-out circles (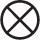) indicate the absence of a condition, and blank spaces indicate an ambiguous state. An ambiguous state means that a condition either exists or does not. A large circle signifies a core condition that exists in both the parsimonious solution and the intermediate solution. A small circle signifies an auxiliary condition (one that exists only in the intermediate solution)*.

#### “Proactive–Overconfidence” in Cross-Industry Mergers

Specifically, concurrent post, innovation, overconfidence, advanced education, and non-financial experience are the core conditions in Conditional Configuration 1a (*PRO*_CP^*^PRO_*R&D*_fz^*^OC_fz^*^MB_EDU^*^~*MB*_*FIN*). This indicates that proactive, overconfident and highly educated CEOs who are ambitious to change the current business environment will overestimate their own ability. This renders them more likely to choose cross-industry mergers when making decisions. This path has the highest explanatory power of the three configurations. Concurrent post and overconfidence are the core conditions in Conditional Configuration 1b (*PRO*_CP^*^PRO_*R&D*_fz^*^OC_fz^*^MB_EDU^*^industry) and Conditional Configuration 1c (*PRO*_CP^*^PRO_*R&D*_fz^*^OC_fz^*^~*MB*_FIN^*^industry). Hence, we named this solution “Proactive–overconfidence.”

#### “Overconfidence-Industry Experts” in Cross-Industry Mergers

In Conditional Configuration 2 (~*PRO*_CP^*^~*PRO*_*R&D*_*fz* ^*^OC_fz^*^MB_EDU^*^~*MB*_FIN^*^~*industry*), overconfidence, advanced education, and traditional industry are the core conditions, while no concurrent post, low innovation, and non-financial experience are the auxiliary conditions. This suggests that CEOs in traditional industries with overconfidence and advanced education tend to choose cross-industry mergers. The path is called “Overconfidence-industry experts.” The chairperson of Guangzhou Development Group is a senior engineer. The company is mainly engaged in the construction and operation of electric power and other infrastructure, which is a traditional industry. In 2019, it acquired Shenzhen Guangfa Electric Power Investment Co., Ltd. for investment management. The total compensation of the top three directors of the company accounted for 33.56% of the total compensation of directors, which was typical for overconfident managers and senior engineers in the industry. The chairperson fits the stereotype of an overconfident manager in a traditional industry.

#### “Innovation–Business Mind” in Cross-Industry Mergers

Conditional Configuration 3 (~*PRO*_CP^*^PRO_*R&D*_fz^*^~*OC*_fz^*^~ *MB*_EDU^*^MB_FIN^*^~*industry*) is named “Innovation–business mind.” This suggests that in traditional industries, managers who are innovative and focus on their own work can make up for their lack of educational background by using their own management knowledge. CEOs are familiar with the capital operation, capital markets, and making decisions prudently. Such managers are more inclined toward cross-industry mergers.

Conditional Configuration 1 and Conditional Configuration 3, “Proactive–overconfidence” and “Innovation–business mind,” can be integrated into proactive CEO. For example, the chairperson of Chongqing Laimei Pharmaceuticals holds a master's degree and has been engaged in biomedical research since university. He has no financial background, although he has served as a director of Jinxing Pharmaceuticals and an executive director of Tibet Laimei Pharmaceuticals. The R&D investment of the company accounts for 11.97% of the operating income, which makes it a highly innovative enterprise. The total compensation of the top three directors accounts for 37.11% of the total compensation of directors. The directors possess enormous power and financial resources, suggesting that they are overconfident managers. In 2014, Chongqing Laimei Pharmaceuticals acquired Heyuan Investment; Chongqing Laimei Pharmaceuticals is a pharmaceutical manufacturing enterprise (C27), while Heyuan Investment Co., Ltd. is a capital market service company (J67). The acquisition of the company incorporated venture capital, industrial investment, equity investment, and investment management of Heyuan Investment Co., Ltd. into the business scope of the company. These departments formed the investment department of the company. In 2008, the chairperson of Furi Group was a senior economist with a junior college degree; he did not have a concurrent post. He belonged to the “Innovation–business mind” manager. The company's R&D investment accounted for 11.97% of the operating income, which made it a highly innovative enterprise. Furi Group was engaged in the textile industry, through an entity called “Towel King.” It entered the photovoltaic industry, and almost considered the photovoltaic industry as the company's first main business. He tried to “save” the home textile industry by creating new growth through cross-industry mergers.

### Conditional Configuration Analysis of Intra-industry Mergers

There are nine configurations with five solutions in Table 7. The consistency of each solution (configuration) and that of the overall solution is higher than the acceptable minimum of 0.75. The consistency of the overall solution is 0.93, and the coverage is 0.52. The analysis shows that 93% of the cases satisfying the nine configurations can lead to intra-industry M&As. Furthermore, the nine configurations can explain 52% of the cases. In this study, the consistency level was adjusted from 0.75 to 0.8 for the robustness test: the research conclusion remained robust.

**Table 7:** Configurations leading to intra-industry mergers.

**Antecedent** **conditions**	**Intra-industry merger (absence of the outcome)**								
	**1a**	**1b**	**2**	**3**	**4a**	**4b**	**4c**	**5a**	**5b**
*PRO_CP*	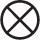	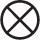	●	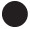		●	●	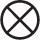	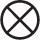
*PRO_R&D_fz*		⊗	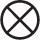		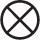	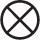		●	⊗
*OC_fz*	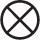	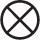		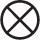	⊗	⊗	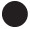		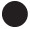
*MB_EDU*	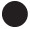	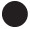	●	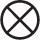	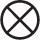	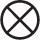	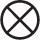	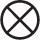	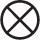
*MB_FIN*	⊗	⊗	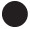	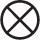	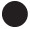	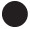	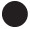	⊗	⊗
*industry*	●		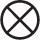	●	●		●	●	
Consistency	0.8255	0.8527	1	0.947	0.9953	0.9955	0.9771	0.9239	1
Raw coverage	0.1518	0.1082	0.1021	0.0658	0.0558	0.0582	0.045	0.0447	0.0532
Unique coverage	0.0676	0.0239	0.1021	0.0432	0.0237	0.0261	0.0253	0.0221	0.0439
Solution consistency	0.9312								
Solution coverage	0.5168								
Cases coverage	6	5	4	3	2	2	2	2	2

*Solid circles (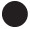) indicate the existence of a condition, crossed-out circles (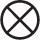) indicate the absence of a condition, and blank spaces indicate an ambiguous state. An ambiguous state means that a condition either exists or does not. A large circle signifies a core condition that exists in both the parsimonious solution and the intermediate solution. A small circle signifies an auxiliary condition (one that exists only in the intermediate solution)*.

#### “Concentration–Industry Experts” in Intra-industry Mergers

Specifically, in Conditional Configuration 1a (~*PRO_CP* ^*^ ~*OC_fz* ^*^ *MB_EDU* ^*^ ~ *MB_FIN* ^*^ *industry*), no concurrent post, non-overconfidence, and advanced education are the core conditions. It shares core conditions with Conditional Configuration 1b (~*PRO_CP* ^*^~ PRO_R&D_fz^*^ ~*OC_fz* ^*^MB_EDU ^*^~ *MB_FIN*). This path has the highest coverage of the nine paths, which can explain about 15% of intra-industry M&A cases. This suggests that CEOs who focus on their own work and industry can make prudent decisions. They are likely to be in favor of intra-industry mergers. Moreover, they are usually not overconfident, nor do they overestimate themselves. We name it “Concentration–industry experts.” Sheng Ji Tang Pharmaceuticals is engaged in the manufacturing of chemical raw materials and chemical products, as well as pharmaceutical manufacturing. The board of Sheng Ji Tang Pharmaceuticals was optimistic about the development prospects of the chemical and pharmaceutical businesses of the Chi Tian Hua Group, especially in the fields of urea, methanol, and pharmaceutical logistics. In 2015, the Chi Tian Hua Group was absorbed in a merger. The president of Sheng Ji Tang Pharmaceuticals, with a bachelor's degree, had no concurrent post or financial experience during his tenure. He was a type of CEO who focuses on his own position and industry. The purpose of the M&A was to strengthen the company's main business and achieve rapid development in the industry.

#### “Low Innovation–Financial Experience” in Intra-industry Mergers

Conditional Configuration 2 (*PRO*_CP^*^~*PRO*_*R&D*_*fz* ^*^MB_EDU^*^MB_FIN^*^~*industry*) can explain 10% of the cases. The analysis indicates that in traditional industries, CEOs lacking innovation but with basic business knowledge will not pursue transformation because the integration risk in a cross-industry merger is higher than that in an intra-industry merger. CEOs with business knowledge are aware of the management problems in the process of M&As; they will thus not blindly implement cross-industry mergers but will prefer intra-industry mergers. The path is named “Low Innovation–financial experience.” In 2015, Shaoxing Wine acquired Zui Zhi Yuan Wine. The chairperson holds a master's degree. In 2014, the R&D investment of the company accounted for 0.32% of the operating income, in line with the low innovation intra-industry M&A profile. The purpose of the M&A was mainly to integrate the land resources of the two enterprises and reserve the necessary land resources for the company's subsequent development of the factory.

#### “Low Education–Deliberation” in Intra-industry Mergers

Regarding Conditional Configuration 3 (*PRO*_*R&D*_fz^*^~*OC*_*fz* ^*^~*MB*_EDU^*^~*MB*_FIN^*^industry), concurrent post, non-overconfidence, low education and non-financial experience are the core conditions. We named it “Low education–deliberation.” Ji Shi Media acquired Jilin Cable Radio and Television Transmission in 2013. The chairperson of the company has a college degree, with no further education. He does not have financial experience, and therefore fits the intra-industry M&A profile of a CEO with low educational background.

#### “Low Education–Financial Experience” in Intra-industry Mergers

Conditional Configuration 4a (~*PRO*_*R&D*_fz^*^~*OC*_*fz* ^*^~*MB*_EDU^*^MB_FIN^*^industry) shares core conditions with Conditional Configuration 4b (*PRO_CP* ^*^~*PRO_R&D_fz* ^*^~*OC_fz* ^*^~*MB_EDU* ^*^ *MB_FIN*) and Conditional Configuration 4c *(PRO_CP* ^*^ *OC_fz* ^*^~* MB_EDU* ^*^ *MB_FIN* ^*^ *industry*). Low education and financial experience are the core conditions, thus named “Low education–financial experience.” The companies that belong to configuration 4 are Chongqing Pharmaceuticals (0.99, 1), the Shanxi Antai Group (0.9, 1), Jiangsu Huaxin Materials (0.79, 1), the Wanhua Chemical Group (0.66, 1), and the Hangzhou Oxygen Generator Group (0.53, 1).

#### “Low Education–Concentration” in Intra-industry Mergers

No concurrent post and low educational background are the common core conditions of Conditional Configuration 5a (~*PRO_CP* ^*^ *PRO_R&D_fz* ^*^~ *MB_EDU* ^*^~ *MB_FIN* ^*^ *industry*) and Conditional Configuration 5b (~*PRO_CP* ^*^~* PRO_R&D_fz* ^*^ *OC_fz* ^*^~ *MB_EDU* ^*^~ *MB_FIN*), thus it is named “Low education–concentration.” The companies that belong to Configuration 5 are Kunming Yunnei Power (0.9, 1), Nanjing Medicine (0.79, 1), the Hunan Aihua Group (0.77, 1), and Henan Zhongyuan Expressway (0.56, 1).

### Connection Between Conditions

Innovation and overconfidence are two important factors in cross-industry M&As. Innovation is the core condition or auxiliary condition for Solution 1(1a, 1b, and 1c) and Solution 2. Overconfidence is the core condition or auxiliary condition for Solution 1 1(1a, 1b, and 1c) and Solution 3. The key factors in intra-industry M&As are low educational background and non-proactiveness. Low educational background is the core condition for Solution 3, Solution 4 (4a, 4b, and 4c), and Solution 5 (5a and 5b). Non-proactiveness is the core condition for Solutions 4 and 5. That is, proactive and overconfident CEOs are inclined toward cross-industry M&As, whereas non-proactive, low-educated CEOs prefer intra-industry M&As. This is different from the traditional quantitative analysis of the symmetry of linear correlation. Moreover, the factors that lead to cross-industry and intra-industry M&As are not single, but multiple concurrent causal relationships.

In cross-industry mergers, “Proactive–overconfidence” occurs in emerging industries, and “Proactive–business mind” occurs in traditional industries. Proactive CEOs prefer cross-industry M&As in both paths. “Overconfidence-industry experts” occurs in traditional industries. It suggests that overconfident CEOs in traditional industries also tend to undertake cross-industry mergers. In the solution of intra-industry mergers, emerging industries appear in the five paths as auxiliary conditions. It is obvious that intra-industry mergers are dominated by emerging industries.

## Discussion

This study examines the influence of CEO proactive personality, overconfidence, background, and industry on M&A decision making. The results of fsQCA show that CEO characteristics and M&A decision-making is multiple concurrent causal relationships, rather than a one-way linear relationship of independent variables and causal symmetry. The results show that there are three configurations for cross-industry mergers and five paths for intra-industry mergers, which is inconsistent with regression analysis symmetry results.

We find that proactive managers who overestimate their capabilities tend to undertake cross-industry M&As. According to the upper echelons theory, previous research has shown that overconfident managers often engage in diversified M&As (Malmendier and Tate, 2008). Malhotra et al. (2018) showed that extraverted CEOs are more likely to engage in acquisitions, and to conduct larger ones, than other CEOs. They are also more likely than other CEOs to succeed in M&As. Moreover, narcissistic CEOs have been examined to invest more in M&A expenditures (Ham et al., 2018). This study adds a new psychological variable for the research on the influence of specific personality traits of executives on M&A cases. From the perspective of proactive personality, our study has extended the research on the influence of proactive personality on strategic decisions. Prior research has suggested that proactive employees had a positive effect on job performance and creativity (Li et al., 2021; Wei et al., 2021). Furthermore, research has also proved that CEO overconfidence is a core condition in cross-industry M&As (Malmendier and Tate, 2008). This study expands on prior research and finds that CEO overconfidence is not the only factor leading to cross-industry M&As. Through the fsQCA method, we find that a proactive personality and overconfidence both influence managers' decisions in emerging industries; they tend to choose cross-industry M&A. In addition, CEOs in traditional industries possessing advanced education and overconfidence tend to choose cross-industry mergers. Consistent with Zhang et al. (2019), we suggested that CEO overconfidence alone does not explain M&A decision-making.

Proactive, overly confident CEOs tend to choose cross-industry M&As. The same is true for proactive CEOs in traditional industries who possess financial experience. Managers with financial experience are better able to use professional financial knowledge to conduct capital operations and deal with crises to some extent (Graham et al., 2013). Custódio and Metzger (2014) have also indicated that CEOs with financial experience will choose aggressive business strategies. In addition, the results have shown that if one proactive manager shows overconfidence, another may tend to be cautious, changing the circumstances. They all choose to pursue cross-industry M&As. Sometimes, managers with different backgrounds, personalities, even working in different industries, may tend to make similar decisions since they evaluate their own management competence and judge industry competition (Chen and Lin, 2018).

Non-innovative CEOs with low education tend to choose intra-industry mergers. Emerging industries dominate such intra-industry mergers. Intra-industry mergers are not exactly the opposite of cross-industry mergers (Fiss, 2011). This is consistent with QCA method characteristics. In practice, intra-industry mergers and cross-industry mergers are not two sides of a coin. CEOs with low education who pay no attention to innovation, but have rich industry experience, tend to choose intra-industry mergers. Wally and Baum (1994) have examined the influence of CEOs' education on management competencies. They found that compared with CEOs with a low education level, CEOs with a high education level had more advantages in information processing. Many innovative and developmental enterprises are mostly led by managers with high education levels. Therefore, CEOs' decisions are consistent with their judgment and competencies. Industry experts can excel at the integration of industries (Chen et al., 2021).

This study also adds perspective on traditional vs. emerging industry conditions, exploring the difference in M&A decision-making between traditional and emerging industries. The most investigated industries have been those of semiconductors, furniture, food, aerospace, and cement in the research on the upper echelons theory (Abatecola and Cristofaro, 2020). In other studies, the industry is usually used as a control variable in M&A studies (Lin et al., 2018). Hambrick and Quigley (2014) considered the industry's condition through the size-weighted mean return on assets. They creatively analyzed CEOs' effect in industries with different grades of discretion. However, our study uses a new type of industry division, which is based on Chinese policy. Our results show that emerging industries dominate intra-industry mergers. Overconfident proactive managers tend to choose cross-industry M&As in the emerging industry sector. At the same time, “Proactive–business mind” and “Overconfidence-industry experts” are two personality types in cross-industry M&As in traditional industries. Emerging industry managers desire integration. As traditional industries are mature, if managers want to make a breakthrough, they tend to enter a new industry to increase operational profits (Lee and Lieberman, 2010).

## Conclusion

This study introduces new conditions for the psychological characteristics of CEOs and deviates from the widely discussed topic of overconfidence and M&As. We follow the upper echelons theory development and use innovation and social capital network to measure CEO proactive personality. On this basis, the study also adds an industry perspective: traditional and emerging industry conditions. The study explores the difference in the choice of M&As between the traditional and the emerging industries.

This research has several theoretical implications for organizations. Firstly, our study has extended the research on the influence of proactive personality on strategic decisions. In previous research, scholars studied the relationship between proactive employees and leadership. For example, Wei et al. (2021) found that proactive personality would energize employees and benefit job performance through decreasing psychological strain under high leader-member exchange. Li et al. (2021) examined the relationship between proactive employees and the organization. They suggested that proactive employees were more likely to engage in multisource information exchange activities with internal and external in the context of social exchange-based employee-organization relationships. Vanwalleghem and Mirowska (2020) studied proactive personality on investor preferences for sustainable investment. The results of their experiment indicated that highly proactive individuals exposed to positive environmental images will remain with the green fund longer than low proactive individuals. Nevertheless, our findings are just the beginning of exploring the influence of proactive personality on the type of M&A. Future study efforts need to expand more on the relationship between proactive personality and other strategic decisions.

Secondly, our study examined the influence of CEO characteristics on intra-industry M&As and cross-industry M&As. Based on the upper echelons theory, strategic decisions will further affect corporate performance (Hambrick, 2007). Scholars have widely studied the influence of CEO characteristics on firms' performance. Wang and Yin (2018) indicated that acquirers paid a lower target premium for education-state deals and the cumulative abnormal announcement returns were positive. Burns et al. (2021) found that acquirer board with supply chain experience was positively related to post-merger operating performance. Therefore, future studies may continue to explore the effect of proactive personality on merger performance. It would also be interesting to study the asymmetric effects of different configurations on post-merger performance (Fiss, 2011).

Thirdly, this study presents a novel perspective on M&A choices between different industries. Previous research regarded industry as a control variable in M&A studies (Lin et al., 2018). According to the division from the Chinese government, our study explores the difference in the choice of M&As between the traditional and the emerging industries. Future studies may explore the black box of the way that personality traits affect decision-making at the industry level (Abatecola and Cristofaro, 2020).

Finally, this study introduces the QCA method to reveal the interaction between different variables and explore the joint effect of characteristics of a CEO on the choice of merger type (Fiss, 2011). Although the framework of the upper echelons theory used here has covered many aspects of socio-demographic features and psychological variables of CEOs including proactive personality, overconfidence, and managerial background. There remain variables that are not discussed. The study does not examine the impact of socio-demographic characteristics (such as CEO age, gender, tenure, and political affiliation) or psychological factors (such as narcissism and extroversion) on M&A decisions. The results show that CEO characteristics and M&A decision-making is multiple concurrent causal relationships, and each characteristic alone does not explain M&A decision-making (Zhang et al., 2019). Therefore, future research directions include exploring the interaction of multiple socio-demographic features and psychological variables on management decision-making. An additional research topic is exploring M&As and business strategies through interviews and qualitative studies. Then, we may research that how these findings affect CEO type in different M&A situations.

The practical implications of the present study are two-fold. Firstly, due to the uniqueness of Chinese mergers and acquisitions, our samples mainly came from Chinese listed companies. China has an active economic environment, featuring rapid transformation, competitive markets. To perform well in a dynamic and uncertain work environment, employees need to be proactive in their job (Crant, 2000). Increasing pressure on CEOs due to fierce competition stimulates greater proactivity. The more proactive the CEO, the more radical the M&A decisions. When making M&A decisions, managers should pay attention to the national environment and economic background, Moreover, they should strive to overcome bounded rationality and cognitive limitations under high pressure (Cristofaro, 2017). Due to the uniqueness of Chinese M&As and economic background, whether our research also applies to companies outside China remains for future research. For instance, when comparing contrasting cultural environments (i.e., American and Chinese), Li and Tang (2013) discovered that CEO hubris was also widely impacted by the beliefs and values at the country-system level.

Secondly, CEO personality and socio-demographic characteristics have been proved to affect the type of M&A decisions. The study's findings could help shareholders understand the role of managers' personality traits in decision-making. To improve the quality of decision-making, the company should pay attention to the characteristics and changes of managers' personality traits in strategy formulation. When making M&A decisions, CEOs must also consider their own competencies, background, and knowledge, judging whether they comprehend the target industry's characteristics and operations (Wally and Baum, 1994; Chen et al., 2021). Managers must attempt successful acquisitions that will positively affect the corporation and reduce the possibility of acquisition failure. Whether M&A achieves industry integration or internal innovation, successful acquisitions provide a steady stream of power for Chinese economic development (Lee and Lieberman, 2010).

## Data Availability Statement

The original contributions presented in the study are included in the article/Supplementary Material, further inquiries can be directed to the corresponding author/s.

## Author Contributions

GY conceived the study and was responsible for the revision of the article. XB was responsible for the research design and the writing of the article. SY was responsible for data collection and analysis. All authors contributed to the article and approved the submitted version.

## Conflict of Interest

The authors declare that the research was conducted in the absence of any commercial or financial relationships that could be construed as a potential conflict of interest.

## Publisher's Note

All claims expressed in this article are solely those of the authors and do not necessarily represent those of their affiliated organizations, or those of the publisher, the editors and the reviewers. Any product that may be evaluated in this article, or claim that may be made by its manufacturer, is not guaranteed or endorsed by the publisher.
